# An In Vitro Model of Angiogenesis during Wound Healing Provides Insights into the Complex Role of Cells and Factors in the Inflammatory and Proliferation Phase

**DOI:** 10.3390/ijms19102913

**Published:** 2018-09-25

**Authors:** Sebastian Beyer, Maria Koch, Yie Hou Lee, Friedrich Jung, Anna Blocki

**Affiliations:** 1 Institute for Tissue Engineering and Regenerative Medicine, Chinese University of Hong Kong, New Territories, Hong Kong, China; sebastian.beyer@cuhk.edu.hk; 2 BioSyM Interdisciplinary Research Group, Singapore-MIT Alliance for Research and Technology, Singapore 229899, Singapore; maria.koch@hdr.qut.edu.au (M.K.); gmsleeyh@nus.edu.sg (Y.H.L.); 3 Centre in Regenerative Medicine, Institute of Health and Biomedical Innovation, Queensland University of Technology, Kelvin Grove, QLD 4059, Australia; 4 Translational ‘Omics and Biomarkers core, KK Research Centre, KK Women’s and Children’s Hospital, Singapore 169857, Singapore; 5 Obstetrics & Gynaecology—Academic Clinical Program, Duke-NUS Medical School, Singapore 169857, Singapore; 6 Institute for Clinical Hemostasiology and Transfusion Medicine, University Saarland, 66421 Homburg/Saar, Germany; dihkf@saarmail.de; 7 School of Biomedical Sciences, Faculty of Medicine, Chinese University of Hong Kong, New Territories, Hong Kong, China

**Keywords:** wound healing, angiogenesis, in vitro model, endothelial cells, pericytes, macrophages, cytokine, sphingolipid

## Abstract

Successful vascularization is essential in wound healing, the histo-integration of biomaterials, and other aspects of regenerative medicine. We developed a functional in vitro assay to dissect the complex processes directing angiogenesis during wound healing, whereby vascular cell spheroids were induced to sprout in the presence of classically (M1) or alternatively (M2) activated macrophages. This simulated a microenvironment, in which sprouting cells were exposed to the inflammatory or proliferation phases of wound healing, respectively. We showed that M1 macrophages induced single-cell migration of endothelial cells and pericytes. In contrast, M2 macrophages augmented endothelial sprouting, suggesting that vascular cells infiltrate the wound bed during the inflammatory phase and extensive angiogenesis is initiated upon a switch to a predominance of M2. Interestingly, M1 and M2 shared a pro-angiogenic secretome, whereas pro-inflammatory cytokines were solely secreted by M1. These results suggested that acute inflammatory factors act as key inducers of vascular cell infiltration and as key negative regulators of angiogenesis, whereas pro-angiogenic factors are present throughout early wound healing. This points to inflammatory factors as key targets to modulate angiogenesis. The here-established wound healing assay represents a useful tool to investigate the effect of biomaterials and factors on angiogenesis during wound healing.

## 1. Introduction

Wound healing is a complex biological process that occurs upon tissue damage. Most of our current knowledge of the fundamental cellular and molecular processes underlying wound healing is derived from animal models. However, although in vivo studies enable the investigation of entire biological processes, they do not allow dissecting these processes into individual aspects. On the other hand, in vitro models allow for a close view on individual factors; nonetheless, these are investigated under isolated conditions. Thus, more complex interactions are often not taken into account [1].

The incomplete understanding of the underlying mechanisms of wound healing under normal and pathological conditions impairs the development of successful approaches for the treatment of nonhealing wounds, reduction of scar formation, and foreign body reaction against biomaterials [2,3].

One fundamental process during wound healing and histo-integration of a biomaterial is the neovascularization [4]. Already during the inflammatory stage, many pro-angiogenic factors are produced [5]. Upon initiation of the proliferation phase, endothelial cells proliferate and migrate into the wound bed and a new vasculature is formed. The vascular bed exceeds the capillary density of normal tissue many fold. During the remodeling phase, many endothelial cells undergo apoptosis and the newly formed vessels regress [6]. Interestingly, the extent of vascularization and the outcome of the wound healing process are believed to be decided at early stage and orchestrated by macrophages [7].

Macrophages are highly heterogeneous and have the ability to polarize towards any phenotype in a wide spectrum with the two extrema M1 and M2 [8]. M1 macrophages are strongly pro-inflammatory and are present at an early stage of wound healing; M2 macrophages can be immune-modulatory and are often referred to as “wound healing” macrophages. They accumulate towards the end of the inflammatory phase and thereby induce the onset of the proliferation phase. It is currently assumed that various macrophage phenotypes orchestrate tissue regeneration and are thus responsible for nonhealing chronic wound, scar formation, and the foreign body response [9,10].

Current in vivo studies had conflicting observations on the role of M1 and M2 macrophages during wound healing. Some studies claim M2 macrophages to be the main promoters of angiogenesis [11,12], whereas M1 macrophages are inhibiting the formation of new blood vessels [11] or have no effect on angiogenesis [12]. Some studies even observed M1 macrophages to be the main driver of endothelial sprouting in vivo [13]. This clearly indicates that in vivo models are not able to sufficiently dissect the different roles of macrophage phenotypes in angiogenesis.

In order to deepen the current understanding on angiogenesis during wound healing, we developed a wound healing model that allows studying angiogenesis in the inflammatory and proliferation phase in vitro. A readily available human monocytic cell line (THP-1) was chosen to generate various macrophage phenotypes. Utilization of a cell line ensured a decreased variability, as we experienced with monocytes from different donors [14]. Sprouting endothelial cells and pericytes were exposed to polarized M1 and/or M2 macrophages in vitro and thus to a microenvironment comparable to the inflammatory or proliferation phase, respectively.

## 2. Results

Endothelial sprouting is an early stage of angiogenesis, during which endothelial cells secrete proteases that degrade the surrounding extracellular matrix (ECM). This process enables proliferation and migration of endothelial cells towards an angiogenic stimulus forming solid endothelial sprouts. To emulate this process in vitro, endothelial cells were seeded as cell spheroids into a collagen I gel and induced to sprout. As a result, defined sprouts grew into the surrounding matrix and could be quantified. As pericytes are understood to play an important role during angiogenesis [15,16], they too were optionally incorporated into the spheroid at a ratio 1:15 (Pericyte:endothelial cell). The optimal seeding ratio was determined earlier [17].

Sprouting endothelial cells were exposed to different macrophage phenotypes in order to provide a microenvironment comparable to that during the inflammatory and proliferation phase of wound healing.

**Figure 1:**
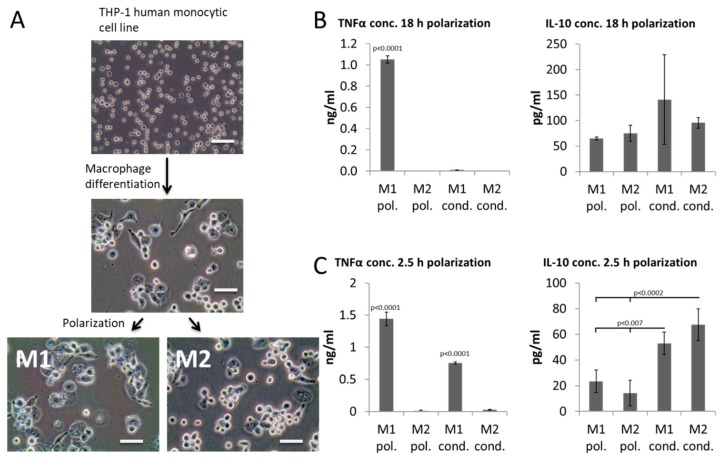
Macrophage polarization. (**A**) Microscopic images depicting morphology of cells at different stages of the polarization protocol. (**B**,**C**) ELISA results for pro-inflammatory TNF-α and immune modulatory IL-10 for polarization (pol.) and conditioned (cond.) media of M1 and M2 macrophages after 18 h (**B**) or 2.5 h (**C**) of polarization. Scale bar: 50 µm. Results are representative for 4 independent runs (*n* = 4 for each condition). They are displayed as mean ± standard deviation.

For this purpose, a human monocytic cell line (THP-1) was differentiated into macrophages and then polarized towards M1 and M2 for 18 h using standard protocols (Figure 1A), after which the polarization medium (M1 pol. and M2 pol.) was collected. To confirm a persisting polarized macrophage phenotype, fresh media (lacking polarization agents) was placed into cultures of polarized macrophages after 18 h to allow subsequent analysis of conditioned media (M1 cond. and M2 cond.). Polarization (M1 pol. and M2 pol.) and conditioned media (M1 cond. and M2 cond.) were analyzed for the presence of TNFα and IL-10 by ELISA (Figure 1B,C) to confirm the anticipated macrophage phenotype.

As expected, M1 polarized macrophages secreted high levels of pro-inflammatory cytokine TNFα and moderate levels of immune modulatory IL-10 into the polarization media (Figure 1B). Surprisingly, the high levels of TNFα could not be detected in the conditioned media of these M1. Thus, TNFα was secreted in an early burst upon polarization and most of the TNFα accumulated in the polarization media and not in the conditioned media. In contrast, M2 polarized macrophages had no detectable levels of TNFα and secreted continuously IL-10.

Since cells resembling true M1 macrophages, thus secreting pro-inflammatory cytokines during the functional assay, needed to be utilized, the polarization protocol was adapted. Macrophages were polarized for 2.5 h only, after which the to-be-conditioned media was added to the cultures (Figure 1C). As a result, TNFα continued to be released upon media change and could now be detected in the polarization and conditioned media of M1 macrophages. The highest levels of IL-10 could be detected in conditioned media of M2 macrophages.

**Figure 2:**
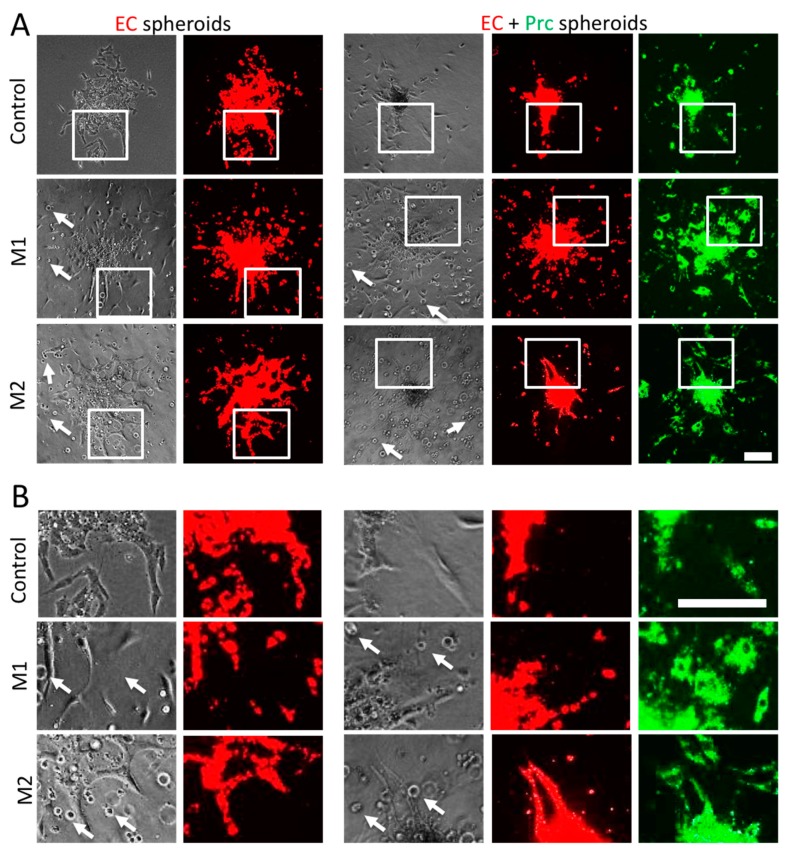
Endothelial cell (EC) sprouting assay in the presence of polarized macrophages. (**A**) Representative images of EC (red) spheroids with and without pericytes (Prcs, green), which were induced to sprout suspended with M1 and/or M2 macrophages. (**B**) Corresponding magnified images from panel A as indicated by white frames. Macrophages appear as small round cells suspended in the collagen I hydrogel and example cells are pointed at by white arrows. Scale bars: 200 µm.

**Figure 3:**
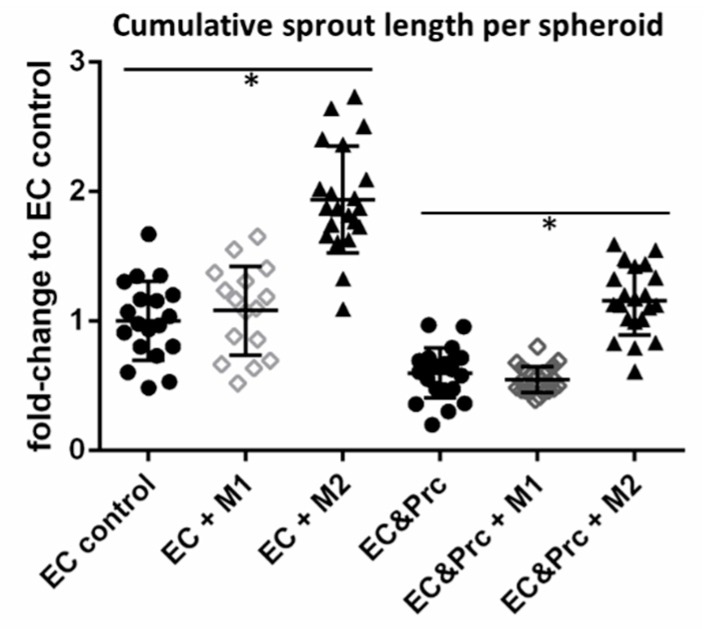
Endothelial cell (EC) sprouting assay in the presence of polarized macrophages. EC spheroids with and without pericytes (Prc) (circle) were induced to sprout suspended with M1(rhombus) and/or M2 macrophages (triangle). Quantification of cumulative sprout length per spheroid normalized to EC control. Results are displayed as scatter plots with mean ± standard deviation; *n* ≥ 16. * indicates a *p*-value < 0.05.

Hence, it was decided to polarize macrophages for 2.5 h and then suspend the polarized macrophages into the collagen I gel of the spheroid sprouting assay (Figure 2 and Figure 3).

Endothelial cells sprouted in all conditions. As reported previously [17], the presence of pericytes within endothelial cell spheroids drastically reduced endothelial sprouting and maintained spheroids with a cell-dense core (Figure 2, controls). Pericytes were located in the cell-dense cores and at formed endothelial sprouts (Figure 2). Macrophages suspended into the collagen I hydrogels appeared evenly distributed in the hydrogels as small round cells (Figure 2, also indicated by white arrows). Suspension of pro-inflammatory M1 macrophages around seeded spheroids did not enhance endothelial sprouting (Figure 2 and Figure 3); however, it induced cell detachment and single-cell migration of endothelial cells and pericytes (Figure 2(M1)). In contrast, presence of M2 macrophages significantly enhanced endothelial sprouting. These endothelial cells formed a network of loose cords and sprouts in the absence of pericytes. However, also in the presence of pericytes continuous, smooth sprouts were formed (Figure 2(M2) and Figure 3).

**Figure 4:**
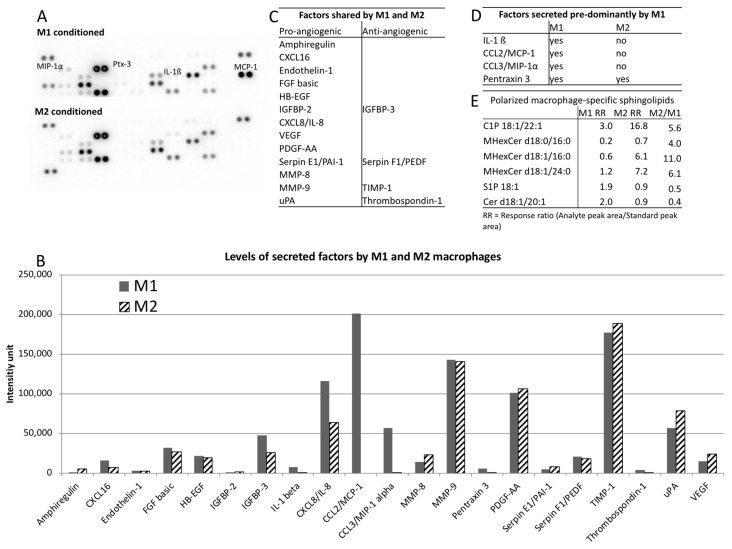
Analysis of macrophage secretome. (**A**) Chemiluminescently developed angiogenesis proteome profiler array membranes incubated with M1 or M2 conditioned media, respectively. (**B**) Signal intensities depicting factors secreted by either M1 or M2 or by both (M1 & M2) as identified proteome profiler array. (**C**) Summary of factors shared by M1 and M2 sorted by their pro- and anti-angiogenic properties. (**D**) Summary of factors secreted solely or predominantly by M1. (**E**) Sphingolipids specific for polarized macrophages as identified by mass spectrometry-based relative quantitation (Sphingolipidomics). Response ratios (RR) were quantified by dividing the analyte peak area/standard peak area. M2/M1 ratio > 2 or < 0.5 with a *p* < 0.05 were defined as sphingolipids with significant differences between M1 and M2.

Macrophages were reported to mediate their effects in a paracrine manner [18]. Therefore, the conditioned media was analyzed for its secretome. An angiogenesis proteome array allowed for a semiquantitative screening of growth factors known to play a significant role during angiogenesis (Figure 4A–E).

Of 46 secreted factors analyzed, 21 (45.7%) were identified to be secreted by M2 and/or M1 and their signal intensities were plotted in a graph (Figure 4A,B, full data available in Supplementary Annex 1). Interestingly both macrophage phenotypes shared a strong pro-angiogenic secretion profile including factors such as bFGF, VEGF and various proteases with some anti-angiogenic factors (IGFBP-3, PEDF, TIMP-1, thrombospondin-1) to modulate the angiogenic stimulus (Figure 4B,C). We did not detect any factor secreted by M2 macrophages that was not present in M1-conditioned media (Figure 4C). However, we detected several factors that were solely (IL-1β, MCP-1, and MIP-1α) or predominantly (Pentraxin 3,10-fold) secreted by M1 macrophages (Figure 4A,B,D).

As it was shown that sphingolipids such as sphingosine-1-phosphate have an immense effect on angiogenesis [19], we also conducted a sphingolipidomics analysis of six sphingolipid classes, namely, ceramide (Cer), ceramide-1-phosphate (C1P), lactosylceramide (LacCer), monohexosylceramide (MHexCer), sphingomyelin (SM), and sphingosine-1-phosphate (S1P) on the conditioned media of M1 and M2 macrophages. To identify sphingolipids associated with macrophage polarization, we defined a M2/M1 ratio as > 2 or < 0.5 with a *p* < 0.05 as significantly different. Of the 88 sphingolipids analyzed, 55 were detected and 6 (10.9%) reached significance. There was increased secretion of C1P d18:1/22:1, MHexCer d18:0/16:0, MHexCer d18:1/16:0, and MHexCer d18:1/24:0 in M2 and an increased secretion of Cer d18:1/20:1 and S1P in M1 (Figure 4E).

## 3. Discussion

A 3D spheroid assay was used to study angiogenic processes in a wound healing environment on a cellular and molecular level. A collagen I hydrogel was employed to provide a three-dimensional microenvironment for the embedded cells. Utilizing collagen I for endothelial sprouting assays is well established [14,17,20] and in contrast to many approaches that utilize fibrin-based hydrogels, is sufficient to stabilize forming sprouts without the addition of protease inhibitors. Indeed, we found that macrophages embedded in the fibrin-based hydrogels digested the hydrogels to an extent that forming sprouts were not stabilized after 24–48 h (unpublished observations). The utilization of a human monocytic cell line instead of freshly isolated monocytes ensured a good reproducibility in this assay. Indeed, THP-1 cells belong to the most common sources to study macrophages and it was shown that major differences between peripheral blood and THP-1-derived macrophages are restricted to resting macrophages. These differences become minimal upon polarization [21]. Partial least square analysis that tested for significant changes in the gene expression of polarized macrophages found that macrophages derived from peripheral blood and THP-1 cells clustered together by phenotype, indicating an overall large overlap in their gene expression profile. Nonetheless, significant differences were found in the expression levels of few genes such as IL-8 [21]. This was confirmed by other studies, reporting that THP-1 cells and their derivates differ from monocytes by e.g., low levels of IL-1β, TNF-α, and lack of IL-8 and IL-10 upon stimulation [22,23]. However, we were able to detect high levels of all these factors. Possible reasons for these differences to previously published findings are an optimized macrophage differentiation protocol including a resting period as established by others [23] and an optimized polarization time period (2.5 h instead of 18 h) ensuring a M1-specific secretion profile during the functional assay. In addition, our analysis of polarized macrophage-secreted sphingolipidomics was independently concordant with a previously published transcriptomics analysis [24]. Further, it was demonstrated that polarized macrophages were not distinguishable by their origin (peripheral blood or THP-1-derived) in terms of their effect on endothelial cell migration [25]. Hence, THP-1-derived polarized macrophages have been well established and are understood to fall within the physiological range of naturally varying macrophages. Taking these data together, we concluded that polarized THP-1-derived macrophages were capable of providing an overall microenvironment sufficiently similar to that presented by primary macrophages suitable for the here-established functional assay.

We were able to confirm that the pro-inflammatory environment, as it was provided by M1, did not inhibit endothelial sprouting, as described in [12], but induced a strong single-cell migration, as it was observed for M1 macrophages previously [25]. In agreement with [11,12], the “wound healing” environment, as provided by M2 macrophages, enhanced endothelial sprouting and induced endothelial network formation.

Cell detachment and single-cell migration of endothelial cells and pericytes away from the seeded spheroid in the presence of M1 suggested that vascular cells migrate in an inflammatory environment without the immediate formation of blood vessels. Only in the presence of M2 macrophages, extensive angiogenesis and the formation of an endothelial tubular network was induced. This emphasized the potential distinct but independently crucial functions of M1 and M2 macrophages in angiogenesis during wound healing, where vascular cells infiltrate the wound bed during the inflammatory phase and incorporate into forming vessels during the proliferation phase.

It was intriguing that, although M1 and M2 macrophages induced such distinctive effects in endothelial behavior, they both exhibited a very similar strongly pro-angiogenic secretion profile. All investigated factors secreted by M2 were also present in M1-conditioned media. These included FGF-2 and VEGF that are believed to be key drivers of angiogenesis during wound healing [26,27]. The only major differences in their tested secretion profile were found to be chemokines associated with the acute inflammatory phase secreted by M1 macrophages. IL-1β, MCP-1, and MIP-1α are all chemoattractants for inflammatory cells such as neutrophils and monocytes/macrophages [28,29,30]. In addition, IL-1β was reported to enhance endothelial cell migration [31] and MCP-1 to recruit vascular smooth muscle cells and mesenchymal stem cells to endothelial cells [32]. Thus, it is likely that these factors will have an essential influence on the cellular motility in the presence of M1 macrophages as observed in this assay. Noteworthy, IL-1β is an activator of sphingosine kinase [33]. It is therefore not surprising to find the highest levels of S1P in M1 polarized macrophages. The controlled balance of ceramide and S1P, two interconvertible cell lipids, is known to direct cellular processes [34]. The presence of both phospholipids in M1-conditioned media indicates that M1 tightly regulate cell cycle arrest, apoptosis, cell survival, and proliferation of surrounding cells [35,36]. Interestingly, M2 macrophages exhibited strongly elevated levels of C1P that is known to inhibit apoptosis and to support cell growth [35]. Thus, M2 also provided a cell survival and proliferatory environment for forming vessels.

The differences observed here in the secretome are in line with a recent lipidomics study to characterize lipid alterations in inflammatory macrophages [36] and recent investigation of lipid profiles between M1 and M2 macrophages [37] and suggest that pro-angiogenic factors are present throughout the early phases of wound healing including the inflammatory phase. Thus, it is likely that the onset of angiogenesis and vessel formation is not initiated by the appearance of pro-angiogenic factors, but by the decline of factors associated with the acute inflammatory phase.

This view on angiogenesis-promoting conditions during wound healing is able to explain many of the observations made by others: Chronic wounds are characterized by a prolonged inflammatory phase and exhibit inadequate angiogenesis leading to impaired wound healing [3,5].

Since pro-angiogenic factors are already present during the inflammatory phase, it is not surprising that applications of pro-angiogenic factors such as VEGF for the treatment of chronic wounds have failed in clinical trials [2,38].

Previously, it was also observed that the level of capillary density in the wound correlates with the extent of the inflammatory response and with the extent of scarring [39]. In our study, the acute inflammatory factors induced infiltration of vascular cells into the wound bed. Therefore, it is likely that enhanced inflammation will lead to an increased accumulation of vascular cells, contributing to a denser capillary network. A higher density of vascular cells in the wound bed will lead to an increased accumulation of apoptotic cells during vessel regression in the remodeling phase. Of note, apoptotic cells have been identified as a new key driver of scarring [39]. This opens new avenues on how macrophages influence scarring during wound healing.

## 4. Materials and Methods

### 4.1. THP-1 Culture

The human monocytic cell line THP-1(ATCC, Manassas, VA, USA, cells were sourced from human peripheral blood with acute monocytic leukemia) was maintained in RPMI-1640 medium (Lonza, Singapore) with 10% FBS (Lonza, Singapore) and 1% Penicillin/Streptomycin(Lonza, Singapore). For macrophage differentiation, 100,000 THP-1 cells per cm^2^ were seeded and supplemented with 100 ng/mL Paramethoxyamphetamin (PMA, Sigma Aldrich, Singapore), for 3 days with additional 3 days resting in maintenance media. THP-1-derived macrophages were polarized by incubation with RPMI-1640 media containing 5% fetal bovine serum (FBS) and 20 ng/mL IFN-γ (Gibco through ThermoFisher, Singapore) and 100 ng/mL Lipopolysaccharide (Sigma Aldrich, Singapore) (towards M1) or 20 ng/mL IL-4 (PeproTech, Rocky Hill, NJ, USA) and 20 ng/mL IL-13 (PeproTech, Rocky Hill, NJ, USA) (towards M2).

### 4.2. Generation of Conditioned Media

Polarized macrophages were washed with phosphate buffered saline (PBS) and 1 mL of EBM-2 (Lonza, Singapore) with 0.5% FBS was added for 24 h. Conditioned media was collected and filtered through a 0.22 µm filter and stored for further analysis. Before conditioned media was mixed 1:1 with fresh EBM-2 containing 0.5% FBS and supplemented with further FBS to reach a final FBS concentration of 2%. It was further supplemented with the EGM-2 BulletKit^TM^ (Lonza, Singapore) to reach fully supplemented EGM-2 (Lonza, Singapore).

### 4.3. ELISA

Successful polarization was confirmed by ELISA for TNFα and IL-10(DuoSet ELISA Development Kit, R&D Systems, Minneapolis, USA) Assay was performed accordingly to manufacturer’s instructions.

### 4.4. Spheroid Sprouting Assay

Human umbilical cord endothelial cells (HUVEC, pooled donor, Lonza Singapore) were cultured in EGM-2 (Lonza, Singapore) and Placenta-derived pericytes (Promocell, Heidelberg, Germany) in Pericytes Growth Medium (PGM, Promocell, Heidelberg, Germany).Cells were labeled live with red or green fluorescence according to manufacturer’s instructions (Sigma Aldrich, PKH67/PKH26). Five hundred HUVEC per well were seeded in EGM-2 containing 2.5 μg/mL methylcellulose into non-adherent round-bottom 96-well plates overnight to form spheroids. When indicated, were added at a ratio of 1:15. Twenty-four spheroids per condition were resuspended in 1.5 mL of EGM-2 containing 2.5 μg/mL methylcellulose and 1 mg/mL naturalized collagen I in a 12 wp well. Pictures were taken over an Olympus IX81 inverted microscope (Olympus, Tokyo, Japan) after 2 days. Cumulative tube length was measured using Fiji software (open source freeware, https://fiji.sc/).

### 4.5. Angiogenesis Proteome Array

The secretion profile of polarized THP-1-derived macrophages was established using an angiogenesis proteome array from (R&D Systems) following the manufacturers protocol. The obtained signals were analyzed via the Protein Array Analyzer plugin for ImageJ by Gilles Carpentier (Faculte des Sciences et Technologie, Universite Paris Est Creteil Val-de-Marne, France).

### 4.6. Mass Spectrometry-Based Sphingolipidomics

Sphingolipids were extracted from conditioned media by a methanol:chloroform mixture under acidic conditions as described previously [40]. Positive ionization mode Liquid Chromatography–tandem mass spectrometry (LC-MS/MS) via Multiple Reaction Monitoring (MRM) on Triple Quadrupole 6460 with electrospray ionization source (Agilent Technologies, Santa Clara, CA, USA) was used for the quantification of sphingolipids as previously described [40].

### 4.7. Statistical Analysis

Statistical relevance was evaluated using one-sided ANOVA and the Tukey post hoc test.

## 5. Conclusions

The here-described functional in vitro assay enabled us to study complex angiogenic processes during wound healing. The current results point to acute inflammatory factors as key inducers of vascular cell infiltration and thus regulators of capillary density in the wound bed and key negative regulators of the onset of angiogenesis. They represent promising targets for new approaches to influence angiogenesis during normal and pathological wound healing. The established assay is robust and represents a promising screening platform for new compounds as well as biomaterials.

## Supplementary Material

Supplementary materials can be found at https://www.mdpi.com/1422-0067/19/10/2913/s1. Supplementary Annex 1: angio membrane quantification.
